# Effects of Athletic Nutritional Supplements on the Human Gut Microbiota: A Narrative Review

**DOI:** 10.3390/nu17193071

**Published:** 2025-09-26

**Authors:** Themistoklis Katsimichas, Anastasia Xintarakou, Charalambos Vlachopoulos, Costas Tsioufis, George Lazaros

**Affiliations:** First Cardiology Department, School of Medicine, Hippokration General Hospital, National and Kapodistrian University of Athens, V. Sofias 114, 11527 Athens, Greece

**Keywords:** gut microbiota, nutritional supplements, protein, branched-chain amino acids, antioxidants, PUFA, athletes

## Abstract

The human gut microbiota constitutes the microbial ecosystem within the human gut. It consists of trillions of mostly prokaryotic microorganisms living in the gut lumen, which have an active metabolic role in the regulation of many host functions, including vitamin synthesis and energy extraction from indigestible fiber. Host diet is the main driver of gut microbial composition and metabolic output. Athletes and athletic individuals often supplement their diet with legal nutritional supplements to enhance performance, especially at the elite level. This review summarizes and critically comments on key evidence of the effects of the most common athletic nutritional supplements on the human gut microbiota, based on the most recent literature. We cover suggested changes in bacterial diversity or the relative abundance of specific bacterial taxa and effects of nutritional supplementation on bacterial metabolism. We focus on the molecular pathways involved, we discuss contrasting results and inconsistencies, and we note limitations and challenges in the field. We conclude with a summary of evidence, proposals, and future directions.

## 1. Introduction

The human gut microbiota, living primarily in the large intestine, is an interdependent community of bacteria, fungi, viruses, and archaea that has co-evolved with the human species [1]. These microorganisms colonize our gut at birth and contribute to essential host-related cellular functions, such as extracting energy from dietary fiber via bacterial fermentation, producing short-chain fatty acids (SCFA) that cross the epithelial barrier to influence human biology, and metabolizing non-dietary chemical compounds [1,2]. The gut microbiota, comprising predominantly anaerobic bacteria, is roughly equivalent in number to human cells and has been the focus of extensive research linking its compositional and metabolic status to various aspects of human health and disease [3,4].

There are essentially two ways by which the microbiota interacts with its host: its mere presence in the gut and its functions, especially the metabolic output of its constituent organisms [4]. The existence of the gut microbiota has long been known to be a crucial factor in the training and maturation of a healthy immune system, as well as a protective factor against colonization of the gut by pathogens. On the one hand, adaptive immunity may have evolved to accommodate and tolerate gut microbial colonization, balancing the benefits of augmented metabolic capacity for humans and the risks of infection [5]. Isolated lymph follicles in the gut develop post-natally based on molecular signals coming from the microbiota, called Microbe-Associated Molecular Patterns (MAMPs), and they constantly sample the gut epithelium above them through the actions of microfold, or M, cells [5]. In this context, CD4+ T_reg_ cells are important for maintaining mutualism and essential for immune homeostasis [6]. On the other hand, pathogens must compete against resilient and long-established gut commensal microorganisms for nutrients and space, facing a strong barrier.

The metabolic activity of the microbiota is also a major influence on human biology, as products of mostly bacterial metabolism can pass through the intestinal barrier and exert effects on the human body. As examples, bacteria synthesize vitamins and harvest energy from complex carbohydrates humans cannot digest, producing SCFA, which have multiple host-related beneficial properties [7,8].

Human diet is the main driver of gut bacterial community structure [9]. Dietary patterns differing in their content (or source) of protein, plant fiber, and other carbohydrates shape different ecosystems in terms of specific microbial abundance, as microorganisms suited to utilizing the main ingested nutrients gain an advantage over others.

Optimal athletic performance relies heavily on nutrition. Many professional and recreational athletes supplement their diet with nutritional supplements permitted by the World Anti-Doping Agency (WADA) to augment performance achieved through training, especially at an elite level, where nutritional intake from food consumption may not be enough.

**Figure 1:**
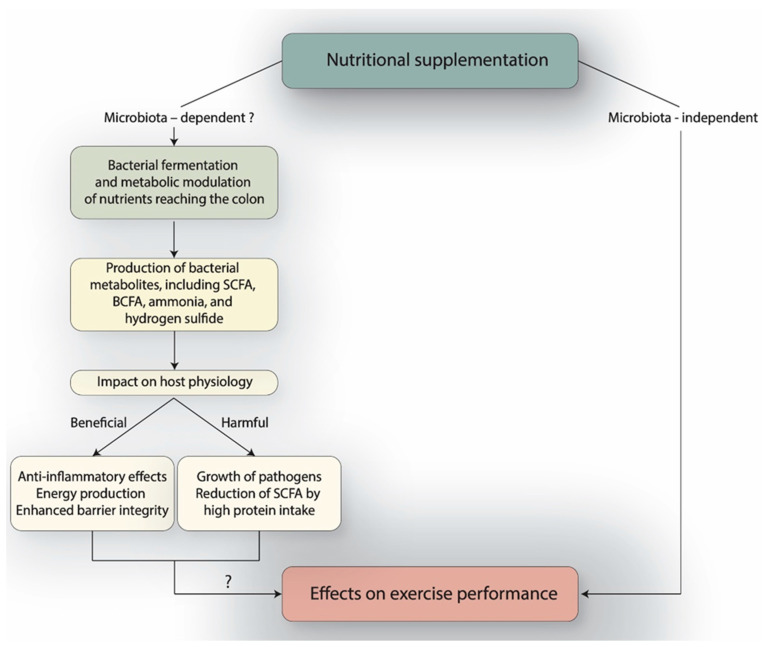
Nutritional supplementation effects on the host and their microbiota. SCFA: Short-Chain Fatty Acids; BCFA: Branched-Chain Fatty Acids.

This narrative review focuses on the effects of the most common athletic nutritional supplements on the gut microbiota: amino acids (arginine, beta-alanine, glutamine, leucine, isoleucine, and valine), antioxidants (vitamins C and E, polyphenols, coenzyme Q10), beetroot juice, betaine, caffeine, citrulline, creatine, iron, probiotics, protein, and PUFA (Polyunsaturated Fatty Acids). We searched PubMed and Cochrane Reviews and scanned the literature for studies on adult humans receiving supplements commonly used by athletes that reported gut microbiota outcomes measured by culture-independent methods, preferably compared to control subjects. We prioritized studies published after 2020 and used the terms “gut microbiota” and the relative supplement name in our query. The exclusion of studies in mouse models was thought appropriate, as the gut microbiota of humans is different in many ways, rendering inter-species comparisons challenging [10,11]. Moreover, germ-free mice colonized with human gut microbiota develop a compromised immune system that may confound results [12], leading to the suggestion that the most valid physiological way to investigate bacteria is in their endogenous hosts [13]. Our review builds upon the foundational work of Zeppa et al. by incorporating recent studies published since 2020 and, more critically, by providing a deeper mechanistic synthesis that contrasts the metabolic impact of different supplement categories (e.g., protein vs. polyphenols) on microbial function [14]. An explanation of methods used in gut microbial analysis and a glossary of terms related to bacterial ecology can be found elsewhere [15]. A summary of the referenced studies, including main findings, is shown in Table 1 and a synopsis of this review is shown in the graphical abstract, Figure 1, and Table 2.

## 2. Athletic Nutritional Supplements

The National Institutes of Health (NIH) Office of Dietary Supplements fact sheet on nutritional supplements for athletic performance provides a thorough overview of claimed effects and critically appraises the relevant literature on the field of athletic nutrition [44]. Based on this document and sources from the International Society of Sports Nutrition, major athletic nutritional supplements were reviewed about their effects on the human gut microbiota (Table 1 and Table 2).

## 3. Amino Acids and Protein

Protein consumption provides amino acids, which muscle cells absorb, using them as building blocks for protein synthesis. Sufficient protein is crucial to optimize the training response to resistance exercise, in terms of increasing muscle mass and strength. Data suggest that athletes require a daily protein intake of 1.2 to 2.0 g/kg of body weight [45,46]. For a male bodybuilder weighing 90 kg, this equates to a maximum of 180 g of protein, which would require a daily consumption of around 690 g of beef or 620 g of chicken, according to nutritional data provided by the European Food Safety Authority (EFSA) [47]. Some evidence supports an even higher daily consumption of >3 g/kg of body weight [48]. Such an intake may be costly or not easily achievable, or it may even have adverse effects on overall health when red meat is concerned, hence the use of protein supplements.

Around 10% of ingested protein passes to the large intestine and is fermented by the gut microbiota [49], which utilizes it as a source of amino acids and energy. Major proteolytic activity in the large intestine is associated with multiple bacterial genera, including *Bacteroides*, *Propionibacterium*, *Streptococcus*, *Fusobacterium*, *Clostridium*, and *Lactobacillus* [50]. A list of gut bacteria and the amino acids they use as metabolic substrates has already been published [51].

Protein consumption positively correlated with microbial alpha diversity in elite rugby athletes [16]. This was one of the few studies actually done with athletes, but its cross-sectional design precludes causality inference, and its specific cohort may hinder generalizability of results to less athletic individuals. Moreover, confounding factors such as calorie restriction and modification of fiber intake may considerably affect proposed changes in gut microbial composition after consumption of high protein diets [52]. In a well conducted study that controlled for fiber intake, microbial composition was not altered, but butyrate concentrations decreased in the high protein diet arm and a modification of gene expression was detected in the rectal mucosa [17]. This shows that high protein consumption will produce a shift in bacterial metabolic pathways towards amino acid degradation. The study was randomized and controlled, inferring causality, and rectal samples added a rare insight into tissue, rather than fecal dynamics. However, the sample was small and consisted of obese individuals only. Similar results regarding compositional stability but significant metabolic shifts have been obtained by others as well, with similar strengths and limitations [18]. In a randomized pilot study of male endurance athletes, a ten-week protein supplementation did not change diversity but altered the relative abundance of individual gut microbial taxa, including the abundance of *Bifidobacterium longum* [19]. This study had a reasonably long follow-up and was randomized and controlled, but both sample and protein dose were relatively small.

Gut microbial metabolism of amino acids found in athletic nutritional supplements is species-dependent and may have beneficial effects for human health. Deamination and decarboxylation of glutamate and alanine produce SCFA (acetate, propionate, and butyrate), while catabolism of branched-chain amino acids (BCAA) leucine, isoleucine, and valine releases branched-chain fatty acids (isovalerate, isobutyrate, isocaproate, and 2-methylbutyrate), which constitute energy sources for gut epithelial cells [53]. Whey protein, the usual form of protein supplementation, is particularly rich in BCAA, thus constituting an excellent source. Glutamine supplementation has been suggested to alter gut microbiota composition, by reducing various genera belonging to the Firmicutes phylum [20]. However, again, small samples and very short study duration challenge the interpretation of results.

Long-term dietary patterns are known to differentially shape the gut microbiota and increased protein intake will favor bacteria evolved to better metabolize amino acids, including genera *Bacteroides*, *Parabacteroides* and *Alistipes* [54]. As mentioned, biochemical routes used by bacteria in the metabolism of amino acids and protein include deamination, decarboxylation, and aromatic transformation. It has been shown that a high protein diet may lead to visceral fat loss mediated by the metabolic actions of the microbiota [18]. Mechanistically, small changes in microbial composition are not so relevant as changes in metabolic activation, as the microbiota is compositionally diverse but metabolically redundant [55]. Therefore, such changes in a certain host may have significant ramifications and high protein diets are shown to alter the gut microbial metabolic landscape. Host-related effects of bacterial amino acid metabolism may include enhanced gut barrier integrity, insulin regulation, and positive modulation of the immune system [56,57,58]. However, not all changes are beneficial to the host: Amino-acid deamination by the gut microbiota produces ammonia, which might negatively affect the gut epithelial barrier [59], whereas degradation of cysteine releases hydrogen sulfide (H_2_S), which could lead to inflammation at high concentrations [60]. The dose–response relationship is likely not linear, and abrupt increases in potentially harmful metabolites could be seen after certain thresholds of protein supplementation.

Inconsistencies between different studies in terms of results are multifactorial and include biological as well as methodological and technical factors. As examples, baseline gut microbiota composition is different in almost every individual, baseline diet may differ in fiber intake, different protein doses affect compositional shifts differently, heterogenous populations vary in transit times, genetics of intestinal enzymatic activity, and intestinal mucosal dynamics, and different durations of intervention may result in compositional and metabolic output variation in the gut microbiota [61,62,63,64].

In summary, protein intake supports muscle growth and strength, but excessive amounts may be impractical or harmful. About 10% reaches the large intestine, where it is fermented by the gut microbiota, influencing its diversity and metabolism. High-protein diets tend to shift microbial pathways toward amino acid degradation, altering metabolite production rather than overall bacterial diversity. Microbial metabolism of amino acids can yield both beneficial SCFA and potentially harmful byproducts like ammonia or H_2_S. Study results are inconsistent because of a combination of biological and technical variation. Arguably, functional changes are more relevant for human health than small compositional changes in microbial taxa. Still, longer follow-up and causal inferences are required to elucidate whether protein supplementation is net beneficial or harmful to host health, or which dose may function as a threshold for microbiota-mediated harm.

## 4. Antioxidants

Antioxidants, including polyphenols, vitamins C and E, and coenzyme Q10, are used for their proposed overall health benefits and capacity to counter oxidative stress and lessen free radical damage to muscles. However, it has been shown that this may not be productive for muscle adaptations to exercise [65,66,67].

Polyphenols are diverse plant-derived chemical compounds with in vitro antioxidant and anti-inflammatory properties that are largely indigestible by humans but can be deconjugated or metabolized by the gut microbiota to bioactive molecules that pass through the intestinal barrier into human blood circulation. This makes the gut microbiota an important mediator of polyphenol-related health benefits. A recent meta-analysis showed that polyphenols generally do not alter bacterial alpha diversity and predictive metagenomics suggested they have no significant effect on metabolic gene family counts [21].

Changes in bacterial abundance after consumption may be polyphenol source dependent. A four-week high consumption of flavanol has been shown to increase concentrations of both *Bifidobacterium* and *Lactobacillus*, generally accepted as bacteria with beneficial effects on human physiology [22]. The cross-over design of this study limits inter-individual variability, but small samples and the lack of a more general metagenomics sequencing approach are notable limitations. Real-time quantitative Polymerase Chain Reaction (qPCR) analysis of targeted bacterial genera has shown that consumption of red wine polyphenols increases concentrations of *Bacteroides* and *Prevotella*, among others [23], while a six-week consumption of wild blueberry powder also increases *Bifidobacterium* [24]. Consumption of a blackcurrant beverage for 4 weeks had no effects whatsoever in the gut microbiota of healthy women in a randomized study [25]. The same was shown for an eight-week xanthohumol supplementation in another randomized study [26]. Consumption of aronia berry polyphenols might cause a change in the relative abundance of butyrate producing *Anaerostipes* [27]. Again, small samples and controversial concepts (enterotypes) are some of the limitations of the studies mentioned above. In a rare case of a metagenomics study, supplementation with inulin and fructo-oligosaccharides led to an increase in *Bifidobacterium* species compared to the placebo arm and numerous compositional shifts within the intervention arm [28]. This was a well-designed study in twin pairs, with promising results.

Vitamin C is a water-soluble vitamin essential for human life and has established antioxidant properties, while Vitamin E (tocopherol) is a fat-soluble antioxidant. Vitamin C supplementation (1 g daily for two weeks, enough to allow passage to the large intestine) was associated with minor gut microbial compositional shifts in a pilot human study, specifically a decrease in the relative abundance of *Bacteroidetes*, *Enterococcus*, and *Gemmiger formicilis* [29]. A four-week daily supplementation of 1 g Vitamin C and daily supplementation of Vitamin E did not alter beta diversity in healthy subjects [30]. Colon-targeted doses and delivery of Vitamin C was a noted strength of these studies, but samples were small. Neither alpha nor beta diversity of the gut microbiota was changed after a four-week daily supplementation of 1 g of vitamin C in another randomized study [31], although there was a suggestion for changes in metabolic gene counts relative to some bacterial metabolic pathways, specifically an increase in counts related to the Entner–Doudoroff (ED) metabolic pathway in the placebo group, which corresponded to measured increase in lipopolysaccharide, and an increase in polyamine biosynthetic pathways also in the placebo group, which corresponded to spermidine levels. Similar results were shown by others, regarding Vitamin E [32]. Evidence from human studies concerning coenzyme Q10 was not found.

Although polyphenols do not seem to change the overall compositional pattern of the gut microbiota, specific changes may have some effect on the host. *Bifidobacterium*, *Lactobacillus*, *Bacteroides*, *Prevotella*, and *Anaerostipes* mentioned above are all linked to multiple host-related benefits. As an example, acetate produced by various species of *Bifidobacterium* protects against pathogenic colonization [68]. It is not entirely clear why changes may be polyphenol-source and -dose dependent. The different study designs and the large molecular diversity of these compounds are probably important factors, but, overall, these relations are largely unexplored [69]. Strikingly, the pre-existing concentrations of certain bacteria in the gut may play a role in the relative abundance changes seen after polyphenol consumption [70]. Hypotheses have been posited on the molecular pathways responsible for the increase in SCFA seen with polyphenol consumption and include the increase in SCFA-producing bacteria and inhibition of α-amylase and α-glucosidase in the saliva and small intestine, possibly increasing substrate concentration in the large intestine [71]. Evidently, an increase in bacteria producing SCFA contrasts with an increase in bacteria degrading amino acids, like the ones mentioned in the section about protein supplementation. This contrast is explained by the difference in substrate availability.

Further molecular mechanisms underlying the effects of polyphenols on the gut microbiota might include the modulation of the gut environment by influencing the intestinal oxidative milieu, thus selecting for bacteria suitable for the relative changes. Of note, conflicting results exist, and one recent meta-analysis has shown that polyphenols may not alter SCFA production in subjects with overweight or obesity [72]. Other reasons for variation in results depending on phenolic substrate may include the bacteriostatic effects of certain polyphenols and preferential metabolism of polyphenols by microbial strains possessing the necessary enzymatic machinery. More variation between studies, beyond that of substrates used in the intervention, can be explained by technical differences, as analyzed in the section on proteins and the section on methodological limitations.

Lastly, vitamins C and E may exert their bacterial modulating effects by reducing oxidative stress in the gut and balancing the fecal redox potential, presumably favoring strictly anaerobic species [73].

To summarize, antioxidants may counter oxidative stress but could hinder exercise adaptations. Polyphenols are metabolized by gut microbes into bioactive compounds, affecting specific bacterial abundances rather than overall diversity. Effects vary by source and dose, often increasing beneficial genera like *Bifidobacterium* or *Lactobacillus*. Molecular mechanisms of change in the microbiota might include polyphenol-induced alteration of the gut environment. Vitamin C and E are generally linked to subtle metabolic pathway changes.

## 5. Beetroot

Red beetroot (*Beta vulgaris*) is a source of nitric oxide (NO), betalains, and polyphenols and it is frequently used by athletes and exercising individuals for its perceived vasodilating effects. There is little research exploring beetroot effects on the gut microbiota. One study has shown that a two-week consumption does not affect either alpha or beta diversity of the gut microbiota, and that it has a minor effect on the abundance of few bacterial species, assessed by real-time qPCR [33]. Similar results were reported by others, in whose study a statistical model accurately predicted subjects’ groups based on bacterial relative abundances. Species corresponding to *Akkermansia*, *Bacteroides*, *Bifidobacterium*, *Butyricimonas*, *Coriobacteriaceae*, *Dialister*, *Mogibacteriaceae*, *Oscillospira*, *Prevotella*, *Rikenellaceae*, *Roseburia*, and *Ruminococcaceae* were higher in the beetroot juice group, whereas *Anaerostipes*, *Christensenellaceae*, *Erysipelotrichaceae*, *Lachnospiraceae*, and *Phascolarctobacterium* were higher in the placebo group [34]. Both studies were too small to support meaningful results.

The effects of beetroot ingredients, including nitrates and polyphenols, are most probably responsible for its presumed effects on the gut microbiota, but the exact mechanisms have yet to be elucidated. Nitrate provides a substrate for NO-producing gut bacteria [74], which could thrive in its presence, including *E. coli* [75]. *Bifidobacterium*, mentioned above as increasing in the presence of beetroot ingredients, is known to produce NO, but *Akkermansia* and *Bacteroides* species, for example, are not, and their possible increase is not easily explained. Our hypothesis is that their increase may be an antagonistic effect of the decrease in other microorganisms, as studies measure relative, not absolute abundances. Another possibility is that other beetroot compounds outweigh the effects of nitrates and promote increase in such bacteria. NO produced by bacteria in the gut might affect epithelial blood flow and, consequently, the balance between aerobic/strictly anaerobic microorganisms. Variation in trial results could be explained by beetroot formulation, among other methodological issues.

Hence, beetroot probably has little impact on overall microbial diversity, but minor shifts may occur in specific bacterial taxa. Some genera, including *Akkermansia* and *Bifidobacterium*, appear increased with beetroot juice consumption. Mechanisms explaining the impact on the microbiota may involve nitrate-utilizing bacteria or interactions with other beetroot compounds, though findings remain inconsistent and largely unexplained.

## 6. Betaine, Citrulline, Creatine

We searched PubMed and the Cochrane library using the terms “gut microbiota” and the respective names of the above in the Title/Abstract fields but did not find evidence on the effects of these supplements on the human gut microbiota, although it is known that several bacterial genera can degrade creatine [76], and many are associated with betaine and citrulline metabolism. A study on the effects of betaine is underway (NCT06758856).

## 7. Caffeine

Caffeine is consumed by athletes and athletic individuals for its perceived effects on alertness, reaction time, strength and endurance, and feelings of energy. The optimal daily dose has been reported as 3–6 mg/kg of body weight, although most studies have been done with the use of coffee, which includes multiple other compounds that can influence performance [77]. One small cross-sectional study suggested that a caffeine intake of >82.9 mg/d may be related to increased richness of the gut mucosa-adherent microbiota [35]. The tissue samples employed add biological credibility to measured changes, but study design precludes causality. A larger, but still cross-sectional study in Spanish subjects hinted at a small effect in the abundance of bacteria of the *Bacteroides*-*Prevotella*-*Porphyromonas* group in those who consumed more than 45 mL of coffee per day [36].

Mechanistically, caffeine may have direct effects on enzymic activity in the colon [78], but its effect on gut microbial abundances is less clear. Since most studies involve coffee rather than caffeine intake, it is possible that other coffee compounds, such as polyphenols, have a greater effect on the gut microbiota. A special link between coffee consumption and a specific species that thrives in its presence has recently been shown [79]. *Lawsonibacter asaccharolyticus* grows better in vitro in the presence of coffee, its growth in the gut is independent of caffeine, and it has a strong positive correlation with coffee consumption worldwide. The precise molecular mechanisms are not clear but may include inhibition of the growth of other organisms. Other mechanisms by which caffeine and coffee may indirectly alter the gut microbiota include changes in transit time and gut secretions. Differences between trials may be explained by caffeine dose, coffee brewing methods, and more general issues in study design.

In summary, caffeine is widely consumed for performance benefits, though most studies examine coffee, which contains additional compounds. Limited evidence suggests caffeine or coffee may modestly increase microbial richness or alter specific taxa, such as *Bacteroides* and *Prevotella*. Coffee appears particularly linked to *Lawsonibacter asaccharolyticus* growth, independent of caffeine, perhaps by inhibiting the growth of competing organisms. Overall, caffeine’s direct microbial effects remain unclear compared to those of other coffee components.

## 8. Iron

Iron is an integral part of hemoglobin and myoglobin, and its supplements are used for their claimed effect on increasing oxygen uptake and decreasing lactate levels during exercise, although subjects without iron deficiency are not expected to gain any advantage. Gut luminal iron is also an essential nutrient for the gut microbiota, and several pathogenic microorganisms are known siderophiles. However, we could not find well-conducted studies on the effects of iron supplementation on the gut microbiota of healthy adults. There is weak evidence that iron supplementation may be associated with negligible bacterial compositional alterations in middle-aged healthy women [37].

Iron formulation may explain some variation in study results [80]. High iron supplement loads are expected to increase siderophore biosynthesis in siderophile microorganisms, as predicted by this study, perhaps in specific species of the *Escherichia* and *Shigella* genera, which have increased iron demands. Iron supplementation has been postulated to alter microbial metabolic pathways and be toxic to beneficial gut bacteria, possibly by harmful oxidative effects [81]. Therefore, caution is required.

## 9. Polyunsaturated Fatty Acids (PUFA)

N-3 polyunsaturated fatty acids, also known as omega-3 fatty acids, are crucial nutrients for humans and they have been shown, among others, to augment aerobic exercise [82]. The effects of these nutrients on the gut microbiota have not been thoroughly researched. Some evidence exists from a randomized trial that they do not alter alpha or beta diversity of the gut microbiota but may increase abundance of several genera, including *Bifidobacterium*, *Roseburia* and *Lactobacillus* [42]. The randomized, cross-over design and the multiple time points sampling are notable advantages of this study. In another study, the Body Mass Index (BMI) of subjects was a more important parameter for gut microbial composition and supplementation also did not alter alpha or beta diversity [43].

The mechanisms linking PUFA with changes in the gut microbiota are not well understood. They may indirectly affect the microbiota by changing the fatty acid gut pool, but they also probably serve as substrates for phospholipid synthesis in some bacterial species [83], of which the increase in relative abundance can decrease the relative abundance of other species. Gut bacteria can take up dietary fatty acids and funnel acyl-groups into the glycerophospholipid pathway via PlsX → PlsY → PlsC (or via acyl-ACP acyltransferases) [84]. PUFA are also enzymatically converted into oxidized derivatives (microbial oxylipins) acting as signaling molecules [85], influencing bacterial interaction both with other microbes and host cells. As bacteria differ in their ability to metabolize PUFA, their intake may select for microbes with particular lipid-related metabolic pathways, possibly changing overall gut microbiota signaling networks and influencing host metabolism. Inconsistencies between published studies may be the result of differences in dose, preparation and combination of PUFA.

## 10. Probiotics

Probiotics are “live microorganisms that, when administered in adequate amounts, confer a health benefit on the host” [86]. They are not nutritional supplements in the literal sense, as they do not contain nutrients. It is expected that administration of a probiotic may increase the relative and absolute abundance of the microorganism(s) involved in the gut of the host, during the time they receive it, and that it may be associated with changes in the relative abundance of other microorganisms. Because probiotics are widely used and promoted as health enhancers, we investigated possible effects they may have on the gut and the gut microbiota of athletes or exercising individuals. Generally, probiotic effects are strain-specific and conditional upon continued delivery, more often reflecting temporary enrichment and functional interactions with resident microbes than gut colonization [87,88,89]. It should be noted that, out of a myriad of claims, the European Union (EU) has approved and accepted only one health claim for a probiotic, the lactose digestion improvement claim for *Lactobacillus delbrueckii* subsp. *bulgaricus* and *Streptococcus thermophilus* in yogurt and fermented milk (EU Register of health claims; Commission Regulation 432/2012).

In a study on trained male endurance athletes, a probiotic compound was shown to positively affect intestinal permeability after 14 weeks of use. This was inferred by a reduction in zonulin in the feces [38]. The authors did not measure gut microbiota abundances. However, they did include athlete-relevant endpoints and used a randomized design. In another study involving recreational triathletes, a combined probiotic/prebiotic/antioxidant formulation was shown to reduce endotoxin blood levels both pre- and post-race. However, when only the combination of the probiotic/prebiotic was given, there was no significant change in endotoxin levels. This implies that only the antioxidant actions were valuable in this case. Similarly to the previous study, the authors did not measure microbiota abundances in their subjects [39]. Other studies have shown that probiotic supplementation may change the abundance of gut bacteria other than those contained in the compound in the gut of the host, possibly through antagonistic or synergistic effects [40,41].

Probiotic supplementation is generally thought to be beneficial for the host immune system, chiefly through the enhancement of SCFA production, which have strong anti-inflammatory properties, gut barrier reinforcement by effects on the tight junctions, increases in mucin expression and secretion by goblet cells, and positive effects on the regulation of T_reg_ cells [90,91]. A study from China including over 25,000 subjects with a ~1:1 sex ratio, who were not sedentary, has shown that intake of foods containing medium levels of live microorganisms (10^4^–10^7^ CFU/g) negatively correlates with the systemic immune-inflammation index [92], although specific associations were not present with foods containing even higher concentrations. Other effects may include improvement in the immune function by activating CD8+ and CD4+ lymphocytes, increases in natural killer (NK) cell activity, a reduction in pro-inflammatory interleukin (IL)-12, IL-6, and IL-4, and increases in anti-inflammatory IL-10 [93]. The effects may subside after discontinuation [93]. Mechanisms affecting the microbiota by probiotic supplementation are centered on the intake of specific strains in large amounts, which may or may not colonize the gut but can cause change by producing metabolites and interacting with the epithelium and the host immune system. Inconsistencies between studies might be explained by differences in strain, dose, formulation, and baseline gut microbial composition, among other methodological issues.

In summary, there is some evidence from randomized studies that probiotics might be associated with changes in the gut microbiota and favorable health outcomes concerning the gut functions of athletic individuals taking them. A multitude of studies also proclaim to offer evidence of the value of probiotics in enhancing athletic performance, possibly through anti-inflammatory properties and positive effects on host metabolic pathways, among others [94]. Significant challenges, including heterogeneity in probiotic selection and dosage as well as outcome measures, hinder interpretation of results [95]. Although some evidence exists on the beneficial effects of probiotics on the host immune system, no relative health claim is currently accepted by European Authorities.

## 11. Limitations and Challenges

Many studies referenced in this review suffer from small samples, heterogenous populations, and methodological limitations in terms of gut microbial analysis. These include targeting different regions of the 16S rRNA gene, which precludes comparisons between studies, lack of data on beta diversity analysis, failure to correct for multiple comparisons, and lack of qPCR data on bacteria with changes in relative abundance. Analyzing different hypervariable regions of the 16S rRNA gene, of which there are 9 in total, introduces systematic bias when one compares different studies, because it affects taxonomic resolution by sequencing [96,97]. A failure to correct for multiple comparisons when simultaneously measuring hundreds of relative abundances between samples invariably increases false positive results, allowing chance to play an unacceptable role in the interpretation of findings [98]. Further, lack of qPCR data in microbiome studies means that a change in the relative abundance of a certain taxon cannot be corroborated by actual measurements and may as well be the consequence of a change in the relative abundance of another taxon, as relative abundances are compositional in nature and always total 100% for each sample.

Some studies in the literature were screened and excluded not because of a flawed design (e.g., a non-randomized design), but because of the use of currently outdated and erroneous statistical tools. An example is the Wilcoxon non-parametric test, which is no longer considered a robust tool for microbiome studies.

Discrepancy in results between studies are likely due to technical issues such as those mentioned above, as well as differences in diet, sex and genetic/geographic background of subjects. Indeed, subject group heterogeneity in terms of age, sex, and ethnic origin, variations in supplement dose and formulation, baseline gut microbiota composition, baseline host diet, and co-intake of other nutritional supplements probably account for a lot of the variation between studies. Causality cannot always be inferred and only 75% of the studies included in Table 1 were randomized controlled trials. Recent evidence also suggests that recreational physical activity may partly mediate the anti-inflammatory effects of certain microbe-rich diets, further supporting the interconnected role of diet and lifestyle in shaping immune responses [92,99]. Therefore, it is not always possible to disentangle the effects of nutritional supplements and lifestyle in physically active individuals. Finally, contrasting results between distinct categories of nutritional supplementation may be explained by the fact that nutritional supplements act as substrates for gut microbial metabolism and, as such, they may differentially affect the gut microbiota, depending on which species are best suited to their use. For example, protein and polyphenol supplementation may result in differences in SCFA productive output, because they essentially drive bacterial metabolic activity in opposite directions. The exact variations and molecular mechanisms underlying these effects require further elucidation, especially since co-intake of supplements differentially affecting the gut microbiota is common. Overall, standardization of supplement composition, formulation, and dose, stratification of subjects by baseline gut microbiota composition and function, control of baseline dietary patterns, application of a multi-omics (metagenomics, metabolomics, and metatranscriptomics) direction, common technical protocols for analyzing microbiomes, and longer follow-up are unmet needs for future studies.

## 12. Conclusions

Athletic nutritional supplements are commercially available and widely circulated. They are used by both athletes and athletic individuals, as well as non-exercising people, for their (perceived) positive effects on health and exercise capacity. These nutrients reach the lumen of the large intestine and are utilized by the gut microbiota. In some, but not all, cases certain supplements have been suggested to shift the structural and metabolic state of the gut microbial ecosystem. However, definitive results cannot be achieved in most cases. Athletic nutritional supplements exert modest effects on the gut microbiota, which are compound-specific and context-dependent. The claims about athletic nutritional supplements are commonly based on short-term, small-sample, heterogeneous studies, usually in non-athlete populations. Importantly, as has already been mentioned, it is challenging to disentangle the effects of nutrient supplementation from those of exercise in studies who recruit athletes, especially when they do not control for training load, periodization, and diet composition.

Hence, the synthesis and integration of the available evidence are not currently sufficient to propose a solid clinical model for use of available athletic nutritional supplements based on their influence on the gut microbiota. This is evidently clear in the example of probiotics, for which no health claim is accepted by the European Union, because of insufficient data. Based on the results of small randomized controlled trials, it appears that the nutritional supplements presented in this review do not change the overall diversity of the gut microbiota but may alter the abundance of specific genera and affect discreet metabolic pathways. Confounders such as dose, calorie restriction, and co-intake of fiber must always be accounted for. A case could be made for high iron supplementation, which may promote the growth of pathogenic bacteria in the gut. Therefore, iron supplementation without iron deficiency is best avoided. A case could also be made against (very) high protein intake, which may reduce SCFA metabolic output by the gut microbiota. Possible negative impacts could be mitigated by co-intake of adequate amounts of fiber. Long-term effects are generally unknown, and more research is required. Larger and more rigorously designed randomized controlled trials, with the use of up-to-date statistical analysis tools and incorporating both metagenomics and metabolomics of fecal and blood samples, will advance knowledge in this field. Currently, the reviewed nutritional supplements should probably be suggested for their perceived effects on athletic performance, rather than their effects on the gut microbiota.

## Figures and Tables

**Table 1:** Summary of referenced studies. RNA: Ribonucleic Acid; PCR: Polymerase Chain Reaction; COVID: Coronavirus Disease; PUFA: Polyunsaturated Fatty Acids; CFU: Colony-Forming Unit; DHA: Docosahexaenoic Acid.

Authors	Subjects	Study Design	Analysis	Main Findings
Clarke SF et al. [16]	40 male Irish elite rugby players, 46 controls	Cross-sectional. Nutritional data based on food frequency questionnaires.	Amplicon sequencing (16S rRNA analysis).	Athletes consumed more protein than controls, accounting for 22% of energy intake. Protein consumption positively correlated with microbial alpha diversity.
Beaumont M et al. [17]	38 overweight individuals	Randomized, controlled, double-blind, parallel design. Three-week isocaloric supplementation with casein, soy protein, or maltodextrin as a control, corresponding to 15% of total energy intake.	Amplicon sequencing (16S rRNA analysis). Metabolite measurements. Rectal mucosa transcriptome analysis.	Microbial composition not altered. Butyrate concentrations decreased in the high protein diet arm. Modification of gene expression detected in the rectal mucosa by use of cDNA microarrays, with clear clustering of differentially expressed genes in the casein and soya group.
Lassen PB et al. [18]	53 overweight or obese individuals	Randomized, controlled, double-blind, parallel design. Sixteen-week isocaloric protein supplementation or control. Test subjects received protein powder preparation containing 34 g of protein (milk protein and free amino acids), 2 g of fat, and 6 g of carbohydrates (i.e., 75%, 12%, and 13% of total energy content, respectively). Controls received an isocaloric mixture containing only 7.3 g of plant and milk protein, 7.6 g of fat, and 24.5 g of carbohydrates, designed to not alter the overall balance of a conventional diet (i.e., 15% protein, 35% fat, 50% carbohydrate).	Shotgun metagenomics.	Minimal effects on microbial composition. Significant shift towards bacterial amino acid metabolism.
Moreno-Pérez D et al. [19]	24 male cross-country runners	Randomized, controlled, double-blind, parallel design. Ten-week isocaloric protein supplementation or control. Protein powder contained blend of 10 g whey isolate and 10 g beef hydrolysate.	Amplicon sequencing (16S rRNA analysis). Quantitative PCR for selected bacteria. Metabolite measurements.	No changes in microbial diversity; decrease in *Bifidobacterium longum* absolute abundance.
de Souza AZ et al. [20]	33 overweight or obese individuals	Randomized, controlled, double-blind. Two-week isocaloric supplementation with 30 g of L-glutamate or control (30 g of alanine).	Amplicon sequencing (16S rRNA analysis).	Reduction in the relative abundance of several genera of the Firmicutes phylum.
Tian R et al. [21]	451 total subjects	Meta-analysis of 6 interventional studies. Polyphenol, galacto-oligosaccharides, inulin supplementation.	Amplicon sequencing (16S rRNA analysis).	Polyphenol supplementation does not alter alpha diversity or predicted metabolic gene counts.
Tzounis X et al. [22]	22 healthy individuals	Randomized, controlled, double-blind, crossover. Four-week high (494 g) or low (23 g) cocoa-derived flavanol supplementation and cross-over.	Fluorescence in situ hybridization (FISH).	High flavanol supplementation increases *Bifidobacterium* and *Lactobacillus*, decreases *Clostridium*.
Queipo-Ortuño MI et al. [23]	10 healthy male volunteers	Randomized, controlled, crossover. Three 20-day periods of de-alcoholized red wine (272 mL/d), red wine (272 mL/d), or gin (100 mL/d) consumption as a source of polyphenols or control (gin).	Real-time quantitative PCR for selected bacterial genera.	Red wine polyphenol consumption significantly increased the numbers of *Enterococcus*, *Prevotella*, *Bacteroides*, *Bifidobacterium*, *Bacteroides uniformis*, *Eggerthella lenta*, and *Blautia coccoides*–*Eubacterium rectale* groups.
Vendrame S et al. [24]	15 healthy male volunteers	Repeated measure, cross-over. Six-week supplementation of a wild blueberry drink (25 g wild blueberry powder in 250 mL water) or control (250 mL water, 7.5 g fructose, 7 g glucose, 0.5 g citric acid, 0.03 g blueberry flavor, 280 µL Allura red AC 1%, 70 µL brilliant blue FCF 1%).	Real-time quantitative PCR for selected bacterial genera.	Increase in *Bifidobacterium*.
Gillies NA et al. [25]	40 healthy female volunteers	Randomized, controlled, double-blind, crossover. Four-week isocaloric consumption of blackcurrant beverage (containing 308 mg of polyphenols and 151 mg of anthocyanins) or control (containing 22 mg of polyphenols and 7 mg of anthocyanins).	Metagenomics.	No significant changes.
Jamieson PE et al. [26]	27 healthy individuals	Randomized, controlled, triple-blind. Eight-week supplementation of 24 g xanthohumol or control (rice protein).	Amplicon sequencing (16S rRNA analysis).	No significant changes.
Istas G et al. [27]	66 healthy male individuals	Randomized, controlled, double-blind, parallel design. Twelve-week supplementation of aronia berry capsules (75 g berries/116 mg phenolic content or 10 g berries/12 mg phenolic content) or control (maltodextrin).	Amplicon sequencing (16S rRNA analysis).	Increase in the relative abundance of *Anaerostipes*.
Ni Lochlainn M et al. [28]	72 elderly individuals (36 twin pairs)	Randomized, controlled, double-blind. Twelve-week supplementation of inulin and fructo-oligosaccharides or control [test subjects and controls received 3.32 g of branched-chain amino acid protein powder, consisting of l-leucine 1660 mg, l-isoleucine 830 mg, and l-valine 830 mg. The intervention arm supplement also contained 7.5 g of prebiotic (Darmocare Pre^®^, Bonusan, Numansdorp, The Netherlands), which consists of inulin (min. 3.375 mg) and fructo-oligosaccharides (FOS) (min. 3.488 mg). The placebo arm supplement contained 7.5 g of maltodextrin powder].	Metagenomics.	Increase in the relative abundance of *Bifidobacterium*, numerous compositional shifts within the intervention arm.
Otten AT et al. [29]	14 healthy volunteers	Longitudinal, not controlled. Two-week supplementation of 1 g Vitamin C/d.	Amplicon sequencing (16S rRNA analysis).	Decrease in the relative abundance of *Bacteroidetes*, *Enterococci*, and *Gemmiger formicilis*.
Pham VT et al. [30]	96 healthy individuals	Randomized, controlled, double-blind, parallel design. Four-week supplementation of vitamins or control in colon release formulations (vitamin A as 250 µg retinol equivalents/d, vitamin B2 as 75 mg riboflavin/d, vitamin C as 500 mg ascorbic acid/d, vitamin B2 75 mg/d + vitamin C 500 mg/d, vitamin D3 as 60 µg cholecalciferol/d, vitamin E as 100 alpha-tocopherol equivalents mg/d, or placebo as 200 mg/d of microcrystalline cellulose).	Metagenomics. Metabolite measurements.	No changes in beta diversity in the Vitamin C and E arms. Vitamin C increased fecal short-chain fatty acid concentrations.
Sim M et al. [31]	40 healthy volunteers	Randomized, controlled, double-blind. Four-week supplementation of 1 g Vitamin C.	Amplicon sequencing (16S rRNA analysis).	No changes in alpha or beta diversity. Predictive metagenomics indicated increase in the Entner–Doudoroff (ED) metabolic pathway in the placebo group, corresponding to measured increase in lipopolysaccharide, and increase in polyamine biosynthetic pathways also in the placebo group, corresponding to spermidine levels.
Chen B et al. [32]	26 healthy individuals	Randomized, controlled. Twelve-week supplementation of Vitamin E (400 IU D-alpha tocopherols/d) or controls (300 mg grape seed extract and 258 mg polyphenols/d or corn amylodextrin 300 mg/d).	Metagenomics.	No changes in alpha or beta diversity.
Wang Y et al. [33]	18 healthy individuals	Longitudinal, not controlled. Two-week consumption of beetroot juice [30 mL concentrate/d containing betacyanins (114.5 ± 3.6 mg), polyphenols (15.6 ± 0.4 mg) and ~228.5 mg nitrate].	Amplicon sequencing (16S rRNA analysis). Real-time quantitative PCR for selected bacterial strains. Metabolite measurements.	No changes in alpha or beta diversity. Changes in the relative abundance of 13 bacterial genera by the intervention.
Calvani R et al. [34]	25 individuals with long COVID	Randomized, controlled, double-blind. Two-week consumption of beetroot juice (200 mL containing ~600 mg nitrate) or control drink (solution containing 7 g of sugar, 180 mL of water, and 20 mL of beetroot juice equivalent to ~60 mg nitrate).	Amplicon sequencing (16S rRNA analysis). Metabolite measurements.	No changes in alpha or beta diversity. A statistical model accurately predicted groups based on bacterial relative abundances. Species corresponding to *Akkermansia*, *Bacteroides*, *Bifidobacterium*, *Butyricimonas*, *Coriobacteriaceae*, *Dialister*, *Mogibacteriaceae*, *Oscillospira*, *Prevotella*, *Rikenellaceae*, *Roseburia*, and *Ruminococcaceae* were higher in the beetroot juice group. *Anaerostipes*, *Christensenellaceae*, *Erysipelotrichaceae*, *Lachno spiraceae*, and *Phascolarctobacterium* were higher in the placebo group. Shifts also noted in fecal metabolite concentrations.
Dai A et al. [35]	34 individuals	Cross-sectional; caffeine consumption.	Amplicon sequencing (16S rRNA analysis).	>82.9 mg/d of caffeine associated with increased richness of the gut mucosa-adherent microbiota.
González S et al. [36]	147 volunteers	Cross-sectional; caffeine consumption.	Real-time quantitative PCR for selected bacterial taxa.	45–500 mL of coffee daily associated with increase in bacteria of the *Bacteroides*-*Prevotella*-*Porphyromonas* group.
Shearer J et al. [37]	56 female individuals	Retrospective; iron supplementation [no iron, low iron (6–10 mg/d), high iron (>100 g/d) groups].	Amplicon sequencing (16S rRNA analysis).	Negligible differences between no/low and high iron supplementation groups.
Lamprecht M et al. [38]	23 endurance male athletes	Randomized, double-blind, placebo controlled. Fourteen-week supplementation of a probiotic compound (4 g/d, including *Bifidobacterium bifidum* W23, *Bifidobacterium lactis* W51, *Enterococcus faecium* W54, *Lactobacillus acidophilus* W22, *Lactobacillus brevis* W63, and *Lactococcus lactis* W58).	Zonulin measurements in feces, various other blood measurements of cytokines and enzymes.	Zonulin excretion decreased in test subjects compared to controls. No other significant results.
Roberts JD et al. [39]	30 recreational triathletes	Randomized, repeated-measures, double-blind, placebo controlled. Twelve-week supplementation with a probiotic/prebiotic/antioxidant compound, a probiotic/prebiotic compound, or control (strains of *Lactobacillus* and *Bifidobacterium* as a probiotic, fructo-oligosaccharides as a prebiotic, 200 mg of α-lipoic-acid and 300 mg of N-acetyl-carnitine hydrochloride as antioxidants, and corn flour as a placebo).	Plasma endotoxin levels and gastrointestinal permeability assessment.	Endotoxin levels lower only in the group receiving the compound including the antioxidant. Gastrointestinal permeability statistically higher in the placebo group post-race, but not in a clinically significant way.
Axelrod CL et al. [40]	7 healthy volunteers	Randomized, double-blind, placebo-controlled, crossover. Four-week supplementation of a probiotic containing *Lactobacillus salivarius* UCC118 (2 × 10^8^ CFU/capsule) or placebo (corn starch with magnesium stearate).	Metagenomics. Gastrointestinal permeability measurements.	Taxonomic sequencing revealed 99 differentially regulated bacteria spanning 6 taxonomic ranks in subjects receiving the supplement. Supplementation attenuated exercise-induced increase in intestinal hyperpermeability.
Lee MC et al. [41]	88 healthy adults	Randomized, double-blind, placebo controlled. Six-week training program and supplementation of *Lactococcus lactis* subsp. *lactis* LY-66 and *Lactobacillus plantarum* PL-02, or control.	Amplicon sequencing (16S rRNA analysis).	No changes in alpha or beta diversity. Increase in beneficial bacteria, especially a significant rise in *Akkermansia muciniphila* following supplementation with PL-02 and LY-66.
Watson H et al. [42]	22 healthy volunteers	Randomized, open-label, cross-over. Eight-week supplementation of 4 g/d N-3 PUFA.	Amplicon sequencing (16S rRNA analysis).	No changes in alpha or beta diversity. Increased abundance of several genera, including *Bifidobacterium*, *Roseburia*, and *Lactobacillus*, observed with intervention.
Pu S et al. [43]	25 individuals at risk for metabolic syndrome	Randomized, controlled, double-blind, cross-over. Four-week supplementation of 5 dietary oil supplements in 5 phases (60 g/d canola oil, DHA-enriched canola oil, canola oil high in oleic acid, a blend of corn oil/safflower oil, and a blend of flax oil/safflower oil).	Amplicon sequencing (16S rRNA analysis).	No changes in alpha or beta diversity.

**Table 2:** Athletic nutritional supplements and possible effects on the human gut microbiota. Abbreviations as elsewhere. Arrows indicate increase or decrease.

Supplement	Effect on Bacterial Diversity	Relative Abundance Changes	Metabolic/Functional Effects
**Amino Acids and Protein**	No major changes	↓ *Bifidobacterium longum* ↓ Genera in the Firmicutes phylum	↑ Amino acid metabolism ↑ Branched-chain fatty acids ↓ Butyrate ↑ Ammonia, H_2_S (potentially harmful)
**Antioxidants (Polyphenols, Vit. C/E)**	No major changes	↑ *Bifidobacterium* ↑ *Lactobacillus* ↑ *Bacteroides* ↑ *Prevotella* ↑ *Anaerostipes* ↓ *Bacteroidetes* ↓ *Enterococci* ↓ *Gemmiger formicilis*	Polyphenol metabolism → ↑ SCFA Vit. C/E → fecal redox-linked changes
**Beetroot**	No major changes	↑ *Akkermansia* ↑ *Bacteroides* ↑ *Bifidobacterium* ↑ *Roseburia* ↓ *Anaerostipes* ↓ *Phascolarctobacterium*	Possible NO-related effects; inconsistent mechanisms
**Caffeine/Coffee**	Possible ↑ richness	↑ *Bacteroides*–*Prevotella*–*Porphyromonas* group ↑ *Lawsonibacter asaccharolyticus* (independent of caffeine)	Affects transit time and gut secretions May modulate enzymatic activity in the colon
**Iron**	No major changes	Potential ↑ siderophiles (e.g., *E. coli*)	Risk of oxidative damage Pathogenic bacterial growth
**Probiotics**	No major changes	Direct ↑ of supplemented strains; sometimes ↑ relative abundance of other genera not included in the formulation	↓ Intestinal permeability ↑ SCFA Immunomodulation
**PUFA (Polyunsaturated Fatty Acids)**	No major changes	↑ *Bifidobacterium* ↑ *Roseburia* ↑ *Lactobacillus*	May change fatty acid pool in the gut Substrates for bacterial phospholipid synthesis Converted to signaling molecules

## Data Availability

Not applicable.
